# Systemic delivery of a specific antibody targeting the pathological N-terminal truncated tau peptide reduces retinal degeneration in a mouse model of Alzheimer’s Disease

**DOI:** 10.1186/s40478-021-01138-1

**Published:** 2021-03-09

**Authors:** Valentina Latina, Giacomo Giacovazzo, Federica Cordella, Bijorn Omar Balzamino, Alessandra Micera, Monica Varano, Cristina Marchetti, Francesca Malerba, Rita Florio, Bruno Bruni Ercole, Federico La Regina, Anna Atlante, Roberto Coccurello, Silvia Di Angelantonio, Pietro Calissano, Giuseppina Amadoro

**Affiliations:** 1 European Brain Research Institute (EBRI), Viale Regina Elena 295, 00161 Rome, Italy; 2 IRCSS Santa Lucia Foundation, Via Fosso del Fiorano 64-65, 00143 Rome, Italy; 3 Department of Physiology and Pharmacology, University of Rome La Sapienza, Piazzale Aldo Moro 5, 00185 Rome, Italy; 4 Center for Life Nanoscience, Istituto Italiano Di Tecnologia, Viale Regina Elena 291, 00161 Rome, Italy; 5 Research Laboratories in Ophthalmology, IRCCS - Fondazione Bietti, Via Santo Stefano Rotondo, 6, 00184 Rome, Italy; 6 Institute of Biomembranes, Bioenergetics and Molecular Biotechnologies (IBIOM)-CNR, Via Amendola 122/O, 70126 Bari, Italy; 7 Institute for Complex System (ISC)-CNR, Via dei Taurini 19, 00185 Rome, Italy; 8 Institute of Translational Pharmacology (IFT), National Research Council (CNR), Via Fosso del Cavaliere 100, 00133 Rome, Italy

**Keywords:** Alzheimer’s Disease, Β-amyloid, Mouse model, Neurodegeneration, Retina, Tau

## Abstract

Retina and optic nerve are sites of extra-cerebral manifestations of Alzheimer’s Disease (AD). Amyloid-β (Aβ) plaques and neurofibrillary tangles of hyperphosphorylated tau protein are detected in eyes from AD patients and transgenic animals in correlation with inflammation, reduction of synapses, visual deficits, loss of retinal cells and nerve fiber. However, neither the pathological relevance of other post-translational tau modifications—such as truncation with generation of toxic fragments—nor the potential neuroprotective action induced by their in vivo clearance have been investigated in the context of AD retinal degeneration. We have recently developed a monoclonal tau antibody (12A12mAb) which selectively targets the neurotoxic 20–22 kDa NH_2_-derived peptide generated from pathological truncation at the N-terminal domain of tau without cross-reacting with its full-length normal protein. Previous studies have shown that 12A12mAb, when intravenously (i.v.)-injected into 6-month-old Tg2576 animals, markedly improves their AD-like, behavioural and neuropathological syndrome. By taking advantage of this well-established tau-directed immunization regimen, we found that 12A12mAb administration also exerts a beneficial action on biochemical, morphological and metabolic parameters (i.e. APP/Aβ processing, tau hyperphosphorylation, neuroinflammation, synaptic proteins, microtubule stability, mitochondria-based energy production, neuronal death) associated with ocular injury in the AD phenotype. These findings prospect translational implications in the AD field by: (1) showing for the first time that cleavage of tau takes part in several pathological changes occurring in vivo in affected retinas and vitreous bodies and that its deleterious effects are successfully antagonized by administration of the specific 12A12mAb; (2) shedding further insights on the tight connections between neurosensory retina and brain, in particular following tau-based immunotherapy. In our view, the parallel response we detected in this preclinical animal model, both in the eye and in the hippocampus, following i.v. 12A12mAb injection opens novel diagnostic and therapeutic avenues for the clinical management of cerebral and extracerebral AD signs in human beings.

## Introduction

Basic and translational researches have recently focused their attention on the eye as an initial site of extra-cerebral manifestations of Alzheimer’s Disease (AD), a neurodegenerative disorder which has been historically perceived as confined to the brain [1, 2]. In particular, among ocular tissues, the retina is a sensory extension of the CNS which shares many structural and functional features with cerebral tissues, including the presence of neurons, glial cells and a blood barrier characterized by a tight regulation in endothelial cell organization [3].

The two classical cerebral lesions of AD pathology—i.e. amyloid β (Aβ) plaques and NeuroFibrillary Tangles (NFT) comprising hyperphosphorylated tau (ptau) protein—have been described in the eyes of both affected patients and experimental transgenic animal models [4–13] in correlation with an early local activation of inflammatory signaling and a reduction in synaptic contacts [14–17] and with functional impairment of visual abilities [18]. Moreover, other signs of ocular degeneration—such as loss of retinal ganglion neurons, atrophy of nerve fiber layer, thinning of the macular ganglion cell complex, axonal degeneration in the optic nerve, alteration of blood flow rate—reflect, and even anticipate, the hallmarks of AD cerebral deterioration [1, 2, 13, 19]. Higher incidence of age-related macular degeneration occurs in patients with AD [20]. Impaired contrast sensitivity, reduced visual acuity, abnormal spatial vision and motion perception are found in AD subjects in tight correlation with the severity of cognitive and behavioural defects [21–32]. Interestingly, ocular defects can manifest even before the appeareance of clinical signs of dementia [33–37]. Of note, 33% of individuals diagnosed with Mild Cognitive Impairment (MCI), a prodromal stage of AD, have substantial visual motion perception deficits and retinal layer thickness together with microvascular alterations [36, 38–42]. In postmortem retinas of MCI and AD patients, extensive retinal pericyte loss along with vascular platelet-derived growth factor receptor-β deficiency are closely associated with increased retinal vascular amyloidosis and predict the cerebral amyloid angiopathy scores [43, 44]. Visual electrophysiology testing has demonstrated significant differences in pattern electroretinogram (PERG), and pattern visual evoked potential (PVEP) in correlation with the retinal nerve fibre layer (RNFL) thickness between AD subjects and healthy controls [45]. Furthermore, utilizing data from retina to develop novel biomarkers for AD offers unique access for direct and non-invasive imaging of pathological changes occurring in the brain [46, 47]. Advances in retinal imaging and evidence of a positive response to immunotherapy of AD animal models prospect widespread population screening, early diagnosis, monitoring before the disease manifests with irreversible clinical symptoms and, eventually, developing disease-modifying intervention [48].

While there are several evidences for the presence of Aβ and phosphotau in eyes from human and animal AD paradigms, efforts are currently being made to identify and validate additional post-translational modifications of tau occurring in the neurosensory retina during disease progression, especially in view of the findings that tau better correlates with the duration and the severity of cognitive decline [49, 50]. In this framework, whether tau cleavage with generation of toxic fragments contributes to visual deterioration and whether their in vivo immunoneutralization evokes a protective action, on both AD retinal and cerebral neurodegeneration, is still lacking.

Our research group has extensively investigated a 20–22 kDa peptide generated from pathological truncation at the N-terminal domain of tau (aka NH_2_htau) which: (1) is detected in cellular and animal AD models [51]; (2) accumulates at human AD presynaptic terminals and is present in CSF from patients suffering from AD and other related tauopathies [52–54]; (3) negatively impacts on synaptic and cognitive functions, both in vitro and in vivo [55, 56]. More recently we have developed a functional cleavage-specific, monoclonal Antibody (mAb)-named 12A12mAb (formerly CCP-NH_2_-tau antiseum (D_25_-(QGGYTMHQDQ) epitope, phosphorylation-independent state [53])—which in vivo selectively neutralizes this harmful specie(s) without significant cross-reaction with the physiological full-length protein. 12A12mAb, when systemically (intravenously, i.v.) injected into 6-month-old Tg2576 and 3xTg mice—two AD models which respectively express the human Amyloid Precursor Protein (APP)695 with Swedish mutations (K670N-M671L), alone or in combination with MAPT P301L and PSEN1 M146V—markedly alleviates into their hippocampi the characteristic biochemical (tau hyperphosphorylation, Aβ accumulation, activation of pro-inflammatory markers), cognitive (spatial memory and orientation), electrophysiological (Long Term Potentiation, LTP induction) and morphological (spine density) alterations [57].

By taking advantage of this well-established tau-directed immunization protocol, we investigated symptomatic (6-month-old) Tg2576 transgenic mice to assess whether: (i) tau cleavage contributes to altering several biochemical, morphological and metabolic parameters of their retina and vitreous body; (ii) 12A12mAb i.v. delivery is able to exert a protective action on the signs of ocular injury associated with the AD phenotype, as shown to occur for the brain parenchyma. This APPSwe-expressing mouse model was chosen because it displays cerebral Aβ deposition and tau modifications, synaptic dysfunction, gliosis, age-dependent memory deficits along with ocular Aβ and tau pathologies [5, 6, 9, 58], thus representing an ideal model to study the AD-associated changes, both in the retina and in the brain.

Here, we show that: (i) tau protein cleavage at the N-terminal extremity is closely associated with other characteristic neuropathological features of AD into retinas and vitreous bodies of Tg2576 animal model; (ii) these ocular changes—which resemble similar modifications occurring in their brain (i.e. APP/Aβ processing, tau hyperphosphorylation, gliosis, loss of synaptic proteins, microtubule breakdown, mitochondrial energetic deficits, neuronal death)—positively respond to systemic treatment with 12A12mAb. These observations are consistent with the contextual improvement of cognitive functions due to antibody-mediated neutralization of N-terminal truncation in the brains of immunized transgenic animals [57], suggesting that the in vivo treatment with 12A12mAb is able to exert parallel beneficial effects on both cerebral and extra-cerebral manifestations associated with the AD phenotype in this preclinical mouse model.

## Materials and methods

### Animals and ethical approval

All animal experiments were complied with the ARRIVE guidelines and were carried out in accordance with the ethical guidelines of the European Council Directive (2010/63/EU); experimental approval was obtained from the Italian Ministry of Health (Authorization n. 524/2017 PR; Authorization n. 1038-2020-PR).

Heterozygous female Tg2576 mice (Tg-AD) (n = 6–8 per group/treatment), expressing the human Amyloid Precursor Protein (APP) with the Swedish mutation KM670/671NL [59] and their sex-matched wild-type (Wt) littermates (n = 5–6 per group/treatment) were used at 6 months of age. The housing conditions (four or five animals per cage) in pathogen-free facilities were controlled (temperature 22 °C, 12 h light/12 h dark cycles, humidity 50–60%) with ad libitum access to food and water. Animals were examined in their overall health, home cage nesting, sleeping, feeding, grooming, and condition of the fur and body weight throughout the whole study and any gross abnormalities were noted. Genotyping was carried out to confirm the presence of human mutant APP DNA sequence by PCR.

### Immunization scheme

The N-terminal tau 12A12 monoclonal antibody (26-36aa) was produced and characterized by Monoclonal Antibodies Core Facility (MACF) at EMBL-Monterotondo, Rome, Italy (Dott. Alan Sawyer), as previously described in [55]. 12A12mAb was purified from hybridoma supernatants according to standard procedures and its purity was determined using SDS-PAGE and Coomassie staining. In detail, the hybridoma supernatant was precipitated by ammonium sulfate (336 g/l). After precipitation, the solution was centrifuged at 10 000 g for 1 h and the pellet was dissolved in PBS and dialyzed against the same buffer. The solution was centrifuged at 10 000 g for 30 min and loaded on a HiTrap Protein G HP (GE Healthcare) equilibrated with PBS. The column was washed with PBS (5 column volumes). 12A12mAb was eluted with 0.1 M Glycine–HCl, pH 2.7. The fractions containing the antibody were neutralized by 1 M Tris–HCl, pH 9.0, collected and immediately dialyzed against PBS. 12A12mAb concentration was determined by measuring the absorbance at 280 nm. The average yield was 8 mg per liter of cell supernatant. 12A12mAb was ≥ 95% pure and contained ≤ 1 U/mg of endotoxin (LAL Chromogenic Endotoxin quantitation kit; Thermo Scientific).

To minimize experimental variability, all mice were initially grouped according to their body weight (20–25 g) and age and mice from the same litter were finally assigned to different groups. The grouped mice were randomized into: (1) wild-type mice treated with saline vehicle; (2) age-matched Tg2576 mice treated with saline vehicle; (3) age-matched Tg2576 mice treated with 12A12mAb (30 μg/dose). Animals were infused over 14 days with two weekly injections administered on two alternate days to the lateral vein of the tail. The dose and route of immunization were based on previously-published studies by our and other independent research groups using Tg2576 as AD transgenic mouse model [57, 60]. In details, mice were placed in a restrainer (Braintree Scientific), and an inch of the tail was shaved and placed in warm water to dilate veins. After injection via the lateral tail vein, mice were returned to home cages and kept under general observation. Abnormalities in overall health, home cage nesting, sleeping, feeding, grooming, body weight and condition of the fur of animals were noted.

Notably, this immunization regimen was previously demonstrated to successfully deliver in vivo a sufficient amount of biologically-active (antigen-competent) anti-tau antibody to promote the clearance of the deleterious NH_2_htau peptide accumulating into animals’ hippocampus and to significantly alleviate their behavioural, biochemical, electrophysiological and anatomo-pathological disease-associated signs [57].

After the immunization schedule, animals were euthanized with CO_2_ and perfused transcardially with ice-cold phosphate buffered saline (PBS). Eyes were removed, dissected, snap-frozen and stored at − 80 °C until further analyses according to [61, 62].

### Western blot analysis and densitometry

Animals from the three experimental groups (wild-type, naive Tg-AD, Tg-AD + mAb) were sacrificed and retinas and vitreous bodies protein extracts were quantified and analyzed for Western blotting according to [63]. In detail, equal amounts of proteins were subjected to SDS-PAGE 7.5–15% linear gradient or Bis–Tris gel 4–12% (NuPage, Invitrogen). After electroblotting onto a nitrocellulose membrane (Hybond-C Amersham Biosciences, Piscataway, NJ), filters were blocked in TBS containing 5% non-fat dried milk for 1 h at room temperature. Proteins were visualized using appropriate primary antibodies, all diluted in TBS and incubated with the nitrocellulose blot overnight at 4 °C. Incubation with secondary peroxidase coupled anti-mouse, anti-rabbit or anti-goat antibodies was followed by the ECL system development and final visualization with the iBright’s digital camera (Thermo Fisher West Pico Plus, U.S.A.; Amersham, Arlington Heights, IL, U.S.A.). Normalization of vitreous and retina samples was carried with β-actin used as loading control [61]. Final figures were assembled by using Adobe Photoshop 6 and Adobe Illustrator 10 and quantitative analysis of acquired images was performed by using ImageJ (http://imagej.nih.gov/ij/).

The following antibodies were used:

Caspase-cleaved protein (CCP) NH_2_-tau antibody rabbit (D25-(QGGYTMHQDQ) epitope, phosphorylation-independent state) [51, 53, 64]; Tau Antibody (BT2) mouse MN1010 ThermoFisher Scientific; anti-N-tau (45-73aa) DC39N1 mouse T8451 Sigma-Aldrich; Phospho-PHF-tau pSer202^+^Thr205 mouse MN1020 ThermoFisher Scientific; PC1C6 Tau1 Ser-195/Ser-198^−^ epitopes mouse MAB3420 Merck Millipore; anti-pan tau protein HT7 (1-150aa of N-terminus) rabbit sc-5587 Santa Cruz Biotechnology; anti-Aβ/APP protein 6E10 (4-9aa) mouse MAB1560 Chemicon; GFAP antibody (2E1) mouse sc-33673 Santa Cruz; anti-GFAP (clone GA5) mouse MAB360 Millipore; Iba1 antibody (1022-5) mouse sc-32725 Santa Cruz; anti-Iba1 rabbit 019-19741 Wako; NMDAζ1 antibody (C-20) goat sc-1467 Santa Cruz; anti-synapsin I antibody rabbit AB1543P Millipore; anti-synaptophysin antibody (D-4) mouse sc-17750 Santa Cruz; anti-syntaxin 1 mouse S1172 Sigma-Aldrich; anti-SNAP25 antibody (clone SMI 81) mouse 836301 BioLegend; anti-α synuclein antibody (clone 42) mouse 610786 BD Transduction Laboratories; cleaved caspase-6 (Asp162) antibody rabbit 9761 Cell Signaling; anti-choactase antibody (H-95) rabbit sc-20672 Santa Cruz; anti-mAChR M1 antibody (H120) rabbit sc-9106 Santa Cruz; anti-vGLUT1 antibody rabbit 135 302 Synaptic System; anti-vGAT antibody rabbit 131 003 Synaptic System; anti-VDAC/Porin antibody rabbit ab34726 Abcam; Tomm20 antibody (FL-145) rabbit sc-11415 Santa Cruz; cytochrome C (136F3) rabbit 4280 Cell Signaling Technology; acetylated α Tubulin (6-11B-1) mouse sc-23950 Santa Cruz; rat anti tubulin alpha mouse MCA77G Bio-Rad; OPA1 antibody mouse 612606 BD Transduction Laboratories; SOD II (MnSOD, mitochondrial superoxide dismutase) rabbit SOD-110D Stressgen Biotechnologies; anti-β-actin antibody mouse S3062 Sigma-Aldrich; anti-mouse IgG (whole molecule)-Peroxidase antibody A4416 Sigma-Aldrich; anti-rabbit IgG (whole molecule)-Peroxidase antibody A9169 Sigma-Aldrich; donkey anti-goat IgG-HRP antibody sc2056 Santa Cruz.

### Immunofluorescence, Epifluorescent acquisition and integrated optical densitometry

Animals of the three experimental groups (wild-type, naive Tg-AD, Tg-AD + mAb) were sacrificed and eyes were rapidly dissected out. Post-fixed eyes were dehydrated and paraffine included. Sections (5 µm thickness; HM325 rotary microtome; Microm, Rijswijk, Netherland) were produced and placed on BDH slides (Milan, Italy), air-dried and stored at − 20 °C. Dewaxed sections were exposed to quenching (50 mM NH_4_Cl, 5 min), antigen retrieval (0.05% trypsin–EDTA solution, 2 min) and blocking/permeabilizing (1% BSA and 0.5% Triton X 100 in PBS, 30 min) steps, and probed overnight with primary antibody (Caspase-cleaved protein (CCP) NH_2_-tau antiserum (D_25_-(QGGYTMHQDQ) epitope, phosphorylation-independent state [51, 53, 64]) diluted (1:200) in PBS (10 mM phosphate buffer and 150 mM NaCl; pH 7.5). The secondary antibody was Cy2 (green) conjugated species-specific antibody (1:1000; donkey; Jackson ImmunoResearch, Europe Ltd, Suffolk, UK) diluted in PBS. Washing steps were performed in PBS containing 0.05% Tween 20. Nuclear counterstaining was performed with 1 μM DAPI solution (D9542; Sigma-Aldrich, St. Louis, MO, USA). Negative control (isotype) was carried out in parallel with the omission of primary antibody and used for appropriate background subtractions. Serial images were analyzed and selected images were digitally acquired (8-tiff) by NIS software connected to epifluorescent direct microscope (Eclipse Ni; Nikon, Tokyo, Japan). For Integrated optical Density (IntDen), the 8-bit TIFF saved digital images (512 × 512 or 1024 × 1024 dpi; n = 4 sections/slide; × 40/dry 0.75 DIC M/N2) were subjected to single analysis with the ImageJ v1.43 (NIH-http://rsb.info.nih.gov/ij/) and expressed in arbitrary units (A.U.) Values were subjected to statistical analysis (wild-type, n = 4; Tg2576, n = 4; Tg2576 + mAb, n = 4).

### Confocal analysis of microglia and astrocytes

For immunofluorescence experiments, mice of the three experimental groups (wild-type, naive Tg-AD, Tg-AD + mAb) were sacrificed by cervical dislocation, eyes were gently removed and kept in 4% PFA solution. After 16 h, eyes were passed into 30% sucrose solution and, after precipitation, were frozen in isopentane and stored at − 80 °C. Sections (50 µm thickness) obtained by a Leica cryostat were treated for immunofluorescence experiments. In brief, slices were treated for 40 min with a warm solution of antigen retrieval (10 mM Na-citrate, 0.05% Tween 20, pH 6.0, 90 °C) to facilitate the exposure of the antigen (when required) and, then, incubated for 45 min in a blocking solution (3% goat serum and 0.3% Triton X-100 in PBS). Primary antibodies were then incubated overnight at 4 °C in a solution with 1% of goat serum and 0.1% of Triton X-100 at different concentrations (anti-Iba1, Wako #019-19741, 1:300; anti-GFAP, Millipore, #MAB360, 1:200). The day after, slices were left 30 min at room temperature, washed three times in PBS, stained with the fluorophore-conjugated antibody and Hoechst for nuclei visualization for 1 h and, finally, mounted in DAKO (Agilent Technologies, CS70330-2) and assessed by confocal microscope (FV10i, Olympus).

For microglia density analysis, images were acquired by using an inverted confocal laser scanning microscope (FV10i Olympus) with a × 60 water immersion objective and a z-step of 1 µm with slices immunolabeled for Iba1. Image processing was performed by using ImageJ software, in order to obtain a maximal intensity projections of z-series stacks. Confocal images were analyzed to count the number of iba1^+^ cells inside the acquisition fields calculated as number of cells per volume (mm^3^): the number of cells within each acquired field was divided by the area of the slice multiplied by its thickness. The value obtained was multiplied by 10^9^ to get the number of microglia present in a mm^3^ of the slice. Only cells whose cell body and processes were fully included in the slice field were included in the analysis.

To assess astrogliosis, slices labeled for GFAP (Glial-Fibrillary Acidic Protein) were acquired by confocal microscopy. Images were then analyzed by Metamorph image analysis software to obtain a maximal intensity z-projection based on GFAP signal. Astrogliosis was then quantified as fluorescence intensity: the threshold was adjusted to accurately represent the number of GFAP-positive cells and data were expressed as area occupied by fluorescent cells versus total slice area.

### Mitochondrial analysis

- Tissue homogenate preparation.
  For mitochondrial analyses, retinas from three experimental groups were stored at − 80 °C until assayed. The PBI-Shredder, an auxiliary high-resolution respirometry (HRR) Tool, was used to prepare homogenate—in 0.2 M phosphate buffer (pH 8.0)—of frozen tissue specimens, according to [65], with high reproducibility of mitochondrial function as evaluated with HRR by means of Oxygraph-2 k OROBOROS®. Homogenate protein content was determined as in [66].
- Enzymatic activity measurements.
  Citrate synthase (CS) and cytochrome c oxidase (COX) activities were measured by spectrophotometric standard methods [65, 67]. Each assay was performed at least in triplicate by using homogenate retinas subjected to three freeze–thaw cycles to disrupt membranes and expose mitochondrial enzymes.
- Measurement of mitochondrial respiratory chain complex (MRC) activities.
  Complex I–V enzymatic activities were assayed photometrically at 25 °C, as in [67]. Each assay was performed at least in triplicate by using retina homogenates subjected to three freeze–thaw cycles to disrupt membranes and expose enzymes. Homogenate from each tissue sample was suspended in 0.3 ml of the respiration medium (consisting of 210 mM mannitol, 70 mM sucrose, 20 mM Tris/HCl, 5 mM KH_2_PO_4_/K_2_HPO_4_, (pH 7.4), 3 mM MgCl_2_) and subdivided to perform three assays [68], which rely on the sequential addition of reagents to measure the activities of: i) NADH:ubiquinone oxidoreductase (complex I) followed by ATP synthase (complex V), ii) succinate: ubiquinone oxidoreductase (complex II) and iii) cytochrome c oxidase (complex IV) followed by cytochrome c oxidoreductase (complex III).
- Measurement of ATP levels.

Retinas were subjected to perchloric acid extraction as described in [69]. Briefly, tissues were homogenized in 600 μl of pre-cooled 10% perchloric acid and then centrifuged at 14,000*g* for 10 min, 4 °C. The amount of tissue ATP was determined enzymatically in KOH neutralized extracts, as described in [70].

### Data management and statistical analysis

Biochemical data were expressed as means ± standard error of the mean (S.E.M.) and were representative of at least three separate experiments (n = independent experiments). Statistically significant differences were calculated by one-way analysis of variance (ANOVA) followed by Bonferroni’s post-hoc test for multiple comparison among more than two groups. *p* < 0.05 was accepted as statistically significant (*p < 0.05; **p < 0.01; ***p < 0.0005; ****p < 0.0001). All statistical analyses were performed using GraphPad Prism 8 software.

For Integrated optical Density (IntDen), the 8-bit TIFF saved digital images (512 × 512 or 1024 × 1024 dpi; n = 4 sections/slide; × 40/dry 0.75 DIC M/N2) were subjected to single analysis with the ImageJ v1.43 (NIH-http://rsb.info.nih.gov/ij/). IntDen data (mean ± SD per retina field) were calculated, grouped and subjected to statistical analysis.

## Results

### Tau cleavage at the N-terminal domain occurs in retina and vitreous bodies of symptomatic Tg2576 AD mice and is reduced by intravenous (i.v.) delivery of 12A12mAb

Post-translational modifications of tau crucially contribute to brain neuropathology of human tauopathies, including AD [71], but the relationship between the truncation and the disease-associated ocular damage has never been studied. Therefore, based on the similarities described between the visual system and the Central Nervous System (CNS) both in human and rodent experimental models of AD neurodegeneration, we investigated whether: (i) tau cleavage—in particular at its N-terminal extremity—could be detected in eyes of symptomatic Tg2576 mice, as we previously found in the hippocampus; (ii) the systemic delivery of 12A12mAb targeting the pathogenic 20–22 kDa NH_2_htau fragment could represent a valuable therapeutic opportunity to ameliorate the retinal injury, known to be associated with their phenotype [1, 6, 9, 72].

**Fig. 1:**
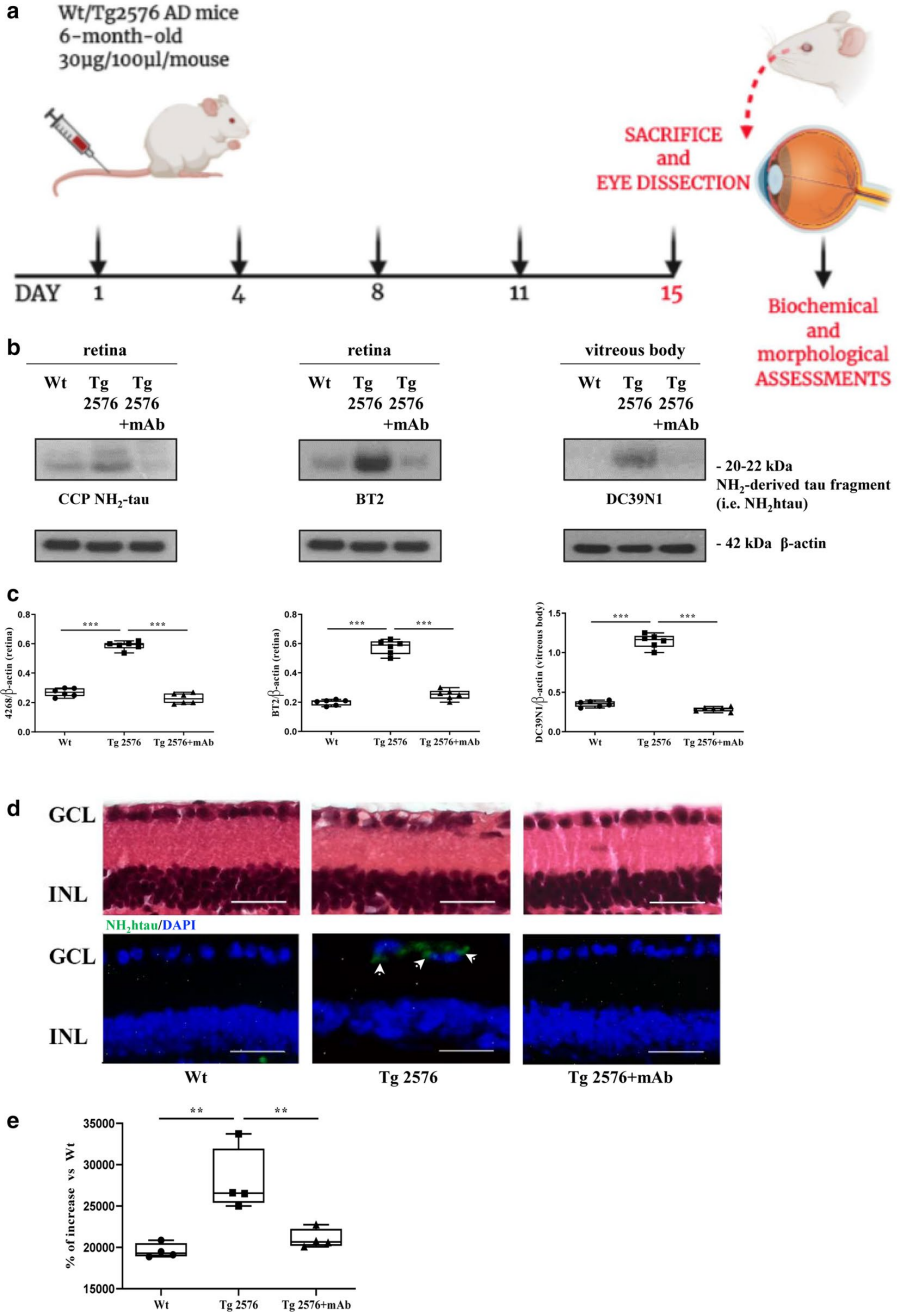
Pathological N-terminal tau truncation occurs in eyes of symptomatic Tg2576 mice and is successfully immunodepleted by 12A12mAb systemic delivery. **a** Study design. 6-month-old Tg2576 Alzheimer’s disease (AD) mice were intravenously (i.v.) injected with 12A12mAb or mouse IgG (isotype control). On day 15, mice were sacrificed and eyes were used for biochemical (Western blotting) and morphological (immunofluorescence) evaluations. Wild type (WT) mice immunized with vehicle (saline) or mouse IgG under the same experimental conditions (antibody dosage, time of treatment, administration route) were used as controls. Picture was assembled by means of Biorender online software (https://biorender.com). Western blotting analyses (**b**) and semi-quantitative densitometric analysis (n = 6) (**c**) carried out on soluble extracts from three experimental groups (wild-type, Tg2576 and Tg2576 + mAb) showing the presence of the NH_2_htau peptide in retina and vitreous body of Tg2576 mice and its 12A12mAb-mediated neutralization following i.v. administration. Filters were probed with three different tau antibodies reacting against different epitopes located around the extremity and middle N-terminal end of protein, including caspase-cleaved protein (CCP)-NH_2_ tau (26-36aa) [51, 64], BT2 (194-198aa) and DC39N1 (45-73aa). β-actin was used as loading control. Arrows on the right side indicate the molecular weight (kDa) of bands calculated from migration of standard proteins. Statistically significant differences were calculated by one-way analysis of variance (ANOVA) followed by Bonferroni’s post-hoc test for multiple comparison among more than two groups. *p* < 0.05 was accepted as statistically significant (*p < 0.05; **p < 0.01; ***p < 0.0005; ****p < 0.0001). D-E: Representative merged panels (**d**) of epifluorescent analysis (n = 4) showing the distribution of the NH_2_htau peptide (green channel) in retinas from three experimental groups (wild-type, Tg2576 and Tg2576 + mAb). Tissues were counterstained with DAPI (blue channel) to aid the visualization of the GCL (Ganglion Cell Layer) and INL (Inner Nuclear Layer). Haematoxylin and eosin stainings were also provided to display the cellular morphology. Histogram (**e**) shows that 12A12mAb immunization is effective in decreasing the NH_2_htau immunoreactivity in transgenic mice (**p < 0.01 versus untreated counterpart, One-way ANOVA, post-hoc Bonferroni test). Values of fluorescent intensity were expressed in arbitrary units (A.U.) Scale bar = 25 µm. Notice that, unlike not-immunized Tg2576, the GCL organization/integrity is well preserved in Tg2576 retinas following 12A12mAb treatment in correlation with a significant diminution in signal of the NH_2_htau

To this aim, we examined and compared, in the eyes of 6-month-old animals from three experimental groups (littermate wild-type, naive/vehicle-treated Tg-AD, Tg-AD + mAb) (Fig. 1a), the pattern of tau truncation at the N-terminal domain along with its in vivo sensitivity to specific antibody-mediated engagement/clearance. Western blotting SDS-PAGE analyses were carried out on soluble homogenates of retinas and vitreous bodies by probing with Caspase-Cleaved Protein (CCP)-NH_2_tau antiserum (D_25_-(QGGYTMHQDQ) epitope, phosphorylation-independent state [51, 64]) followed by semi-quantitative densitometry. As shown in Fig. 1 b, c, we found that the endogenous steady-state expression level of the toxic NH_2_htau peptide was significantly increased in ocular samples from 6-month-old Tg2576 AD mice in comparison to their wild-type littermate controls (***p < 0.0005). This finding was also confirmed with BT2 (194-198aa) and DC39N1 (45-73aa), two other commercial tau antibodies reacting against different epitopes located around the extremity and middle N-terminal end of tau (***p < 0.0005). In line with its aberrant release from cortical synapses [73] and its accumulation in peripheral CerebroSpinalFluids (CSF) from AD-affected subjects [52], this soluble N-terminal truncated tau specie(s) turned out to be present in the vitreous body, an ocular fluid whose protein composition depends on secretion from surrounding tissues (ciliary body and retina) [74]. Notice that, as previously detected in hippocampus [57], the immunoreactivity signal of the toxic NH_2_htau peptide in Tg2576 mice was strongly reduced following 12A12mAb immunization in comparison with their naïve not-vaccinated counterparts (***p < 0.0005).

To further validate these biochemical observations, morphological studies of epifluorescence microscopy, followed by integrated optical densitometric analysis, were carried out for the detection and/or distribution of the NH_2_htau fragment in retinal sections. As shown in Fig. 1d, e, the labeling with Caspase-Cleaved Protein (CCP)-NH_2_tau antiserum (D_25_-(QGGYTMHQDQ) epitope, phosphorylation-independent state [46, 59]) showed a strong increase in the intracellular positivity in AD transgenic mice (**p < 0.01 versus wild-type controls) with a granular, dot-like aspect which appeared to be mainly distributed to the Ganglion Cell Layer (GCL) consisting of retinal ganglion cells and displaced amacrine cells (arrows). These findings are in agreement with previous investigations reporting an apical localization of pathological tau in diseased retina [10, 63, 75]. On the contrary, no staining was clearly detectable either in the superior or in the inferior retinal part of littermate wild-types. Interestingly, a statistically-significant general reduction in signal intensity was found in 12A12mAb-immunized transgenic animals (**p < 0.01 versus untreated counterpart), consistent with results from Western blotting (Fig. 1b, c).

By extending previous studies on the presence of epitope-specific phosphorylation and accumulation of tau in the eyes of AD subjects [15, 76] and transgenic mouse models [6, 63, 77–80], these results show that protein cleavage is a pathological alteration detectable in ocular samples of 6-month-old Tg2576 AD animals, as previously shown for the brain parenchyma [57]. More importantly and consistent with promising results on the clearance of retinal Aβ in an animal model of Age-related Macular Degeneration (AMD) [81], this preclinical study supports the in vivo feasibility of tau-based immunotherapeutic approach—which specifically intercepts the pathologically-relevant species of the protein—as strategy to contrast the eye damage and vision loss occurring in AD development.

### Systemic administration of 12A12mAb mitigates tau hyperphosphorylation and APP/Aβ misprocessing in the retina and vitreous body of Tg2576 AD mice

A significant increase in the immunoreactivity of APP along with the deposition of insoluble Aβ-positive aggregates and pathological site-specific tau hyperphosphorylation have been found in the retinas of aging Tg2576 animals [6, 9, 82] and in human affected subjects [1, 15]. Likewise, changes in the levels of total tau and Aβ1-40/1–42 peptides in vitreous humor from AD patients are clinically predictive of their neuro-cognition state evaluated by Mini-Mental State Exam (MMSE) [76]. Therefore, by SDS-PAGE Western blotting with specific antibodies (AT8/Tau-1 6E10), we further analyzed the soluble ocular homogenates from mouse retina and vitreous bodies to evaluate whether the treatment with 12A12mAb could impact on the AD-like tau hyperphosphorylation and APP/Aβ misprocessing/accumulation.

**Fig. 2:**
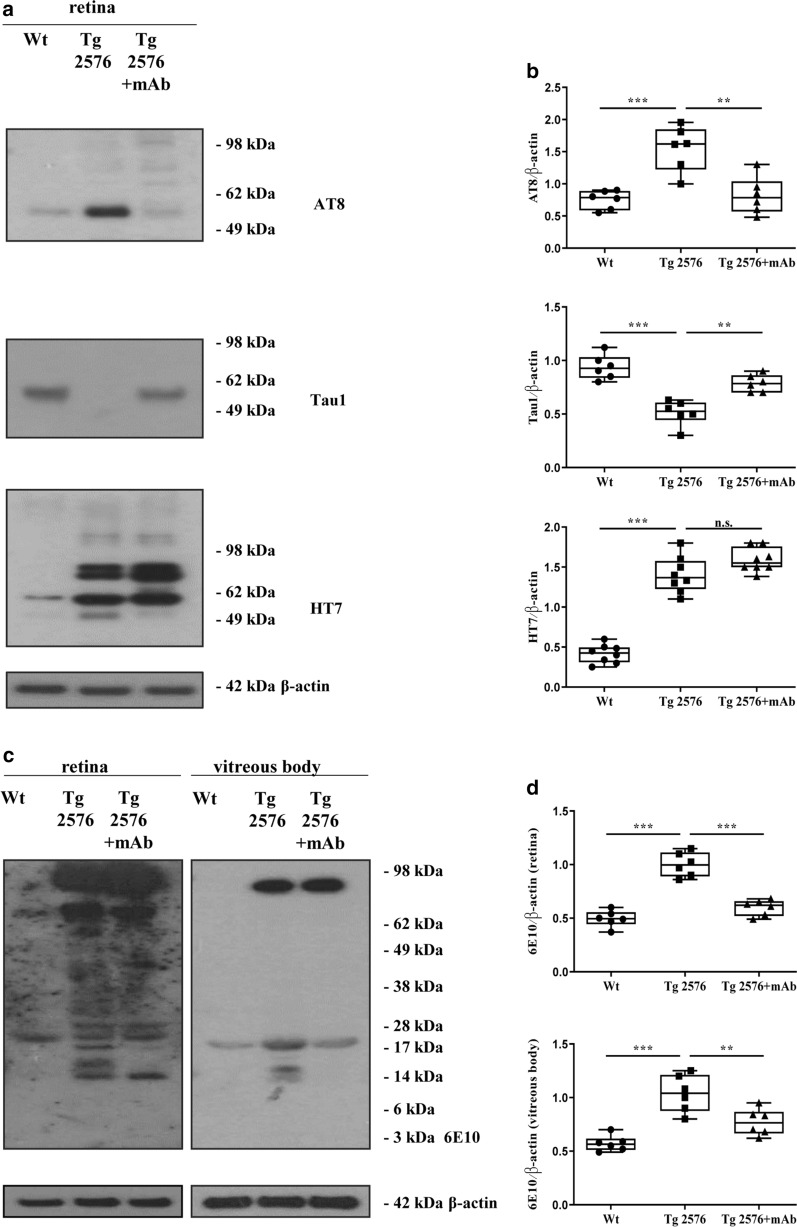
The epitope-specific AD-like tau hyperphosphorylation and APP/Aβ dysmetabolism found in eyes from Tg2576 animals are strongly reduced by 12A12mAb i.v. injection. Western blots (**a**) of soluble retinal homogenates from three experimental groups (wild-type, Tg2576 and Tg2576 + mAb) probed with specific antibody against total (HT7), phospho- (AT8, P + Ser198/Ser202 epitopes) and dephospho- (Tau-1, P-Ser198/Ser202 epitopes) tau protein. Arrows on the right side indicate the molecular weight (kDa) of bands calculated from migration of standard proteins. Semi-quantitative densitometric analysis (n = 6) of all retinal tau isoforms was shown in (**b**) by using β-actin for normalization. Values are from at least three independent experiments and statistically significant differences were calculated by one-way analysis of variance (ANOVA) followed by Bonferroni’s post-hoc test for multiple comparison among more than two groups. *p* < 0.05 was accepted as statistically significant (*p < 0.05; **p < 0.01; ***p < 0.0005; ****p < 0.0001). Western blotting probed with 6E10 (anti-Aβ/APP protein, 4-9aa) (**c**) showing that the immunoreactive bands of Amyloid Precursor Protein (APP)-derived, Aβ-containing processing intermediates were significantly reduced in retina and vitreous body from 12A12mAb-injected transgenic AD animals. Semi-quantitative densitometric analysis (n = 6) (**d**) was calculated by normalizing the smeared signal ranging between 12 and 95 kDa of each lane/sample (the region of interest, ROI) to corresponding β-actin intensities on the same blots. Values are from at least three independent experiments and statistically significant differences were calculated by one-way analysis of variance (ANOVA) followed by Bonferroni’s post-hoc test for multiple comparison among more than two groups. *p* < 0.05 was accepted as statistically significant (*p < 0.05; **p < 0.01; ***p < 0.0005; ****p < 0.0001)

As shown in Fig. 2a, b (upper panel) and in line with results from ocular samples of 3xTg-AD paradigm carrying the human mutations tauP301L/PS1M146V/APPSwe [63], semi-quantitative densitometry of the signal from AT8 mAb (pSer202/pThr205 epitopes) revealed a strong upregulation in the intensity of the 55–70 kDa MW bands from retinas of 6-month-old Tg2576 (***p < 0.0005 versus littermate wild-type controls). This finding fits well with the strong immunoreactivity of AT8-hyperphosphorylated tau described in the brain parenchyma [57, 83–86] and in cross retinal sections from aged (10-month-old) Tg2576 [6] and human AD cases [15]. Strikingly, the 12A12 passive immunization reduced the specific AT8 tau-positive pattern (**p < 0.01 versus naive, saline-treated animals), as we previously reported to occur in the brain parenchyma [57]. Consistently with the upregulation in AT8 tau immunoreactivity of the 55–70 kDa MW band, an inverse decrease in reciprocal signal was detected in Tg2576 mice (***p < 0.0005 versus wild-type controls) and in a 12A12mAb-dependent manner (**p < 0.01 versus naive, saline-treated counterpart) by probing the filter with the complementary Tau-1 antibody (non-phospho Ser198/Ser202 epitopes) [87]. It is noteworthy that AT8 and Tau-1 antibodies specifically stain in a similar reciprocal pattern diseased tau from affected brain areas in late AD subjects [88]. Furthermore, by using an anti-pan tau HT7 mAb (159-163aa of N-terminus) which detects the total tau irrespective of its phosphorylation state, we found a significant elevation/accumulation of all protein isoforms in Tg2576 samples (***p < 0.0005 versus littermate wild-type controls), indicating that the APPSwe mutation induces per se an upregulation of endogenous murine tau protein in retinas of this transgenic animal strain. Interestingly, the steady-state expression level of the 100 kDa MW band, a less predominant tau isoform which is more likely to correspond to the High-Molecular-Weight big tau present only in peripheral neurons [89–91], also slightly increased under pathological conditions. However, as we previously found in the corresponding hippocampi [57], following 12A12mAb injection, the 55–70 kDa MW signals remained largely unchanged when transgenic animals were compared with their not-immunized counterpart (*p* > 0.05), indicating that immunization per se did not aspecifically affect the overall expression of tau.

By probing retinal extracts from animals’ cohorts with the anti-Aβ/APP protein 6E10 (4-9aa) antibody (Fig. 2c, d), an increase in the expression level of APP full length holoprotein along with a prominent heterogeneous ladder of Aβ sequence-containing processing intermediates ranging between 14 and 70 kDa was clearly discernible in 6-month-old Tg2576 AD mice when compared with littermate wild-type controls (***p < 0.0005). As matter of fact a pronounced degradation of full-length overexpressed APP was detectable at 6 months in this AD model, thus extending previous investigations on retinas of 14-month-old aged animals [9]. Consistent with results from their diseased brain parenchymas [57], an overall weaker immunoreactivity pattern was calculated in the same animals’ ocular samples following immunization with 12A12mAb (***p < 0.0005 versus naive not-injected conterpart). Notably, in retinal extracts of Tg2576 as well as in other mutated APP-overexpressing mouse strains, there was only a faint signal for Aβ peptide at the expected apparent electrophoretic mobility of 4 kDa [9]. This is in agreement with the evidence that the 4 kDa Aβ peptide is generated in the peripheral nervous system at a lower extent than in the brain [9, 82], regardless of the local expression of APP and related amyloidogenic processing secretases [74, 92]. Besides, when ocular fluids and vitreous bodies were analyzed by Western blotting we found out a similar but less pronounced 6E10-positive trend in extracellularly-secreted ocular amounts of APP/Aβ-derived immunoreactivity, further confirming the local anti-amyloidogenic effect following 12A12mAb systemic treatment (***p < 0.0005 Tg2576 versus wild-type; **p < 0.01 Tg2576 + mAb versus not-immunized counterpart).

Taken together, these data demonstrate that pathological N-terminal truncation of tau with generation of the toxic 20–22 kDa tau fragment occurring in the eyes of Tg2576 AD is linked to the other two well-established pathognomonic features (AT8 site-specific tau hyperphosphorylation and APP/Aβ amyloidogenic processing) detected in their ocular structures (retina and vitreous body) and in a 12A12mAb-reversible manner, as we previously reported to occur in animals’ hippocampi [57].

### The up-regulation of inflammatory markers in retina of Tg2576 AD mice is relieved following 12A12mAb-based immunization

**Fig. 3:**
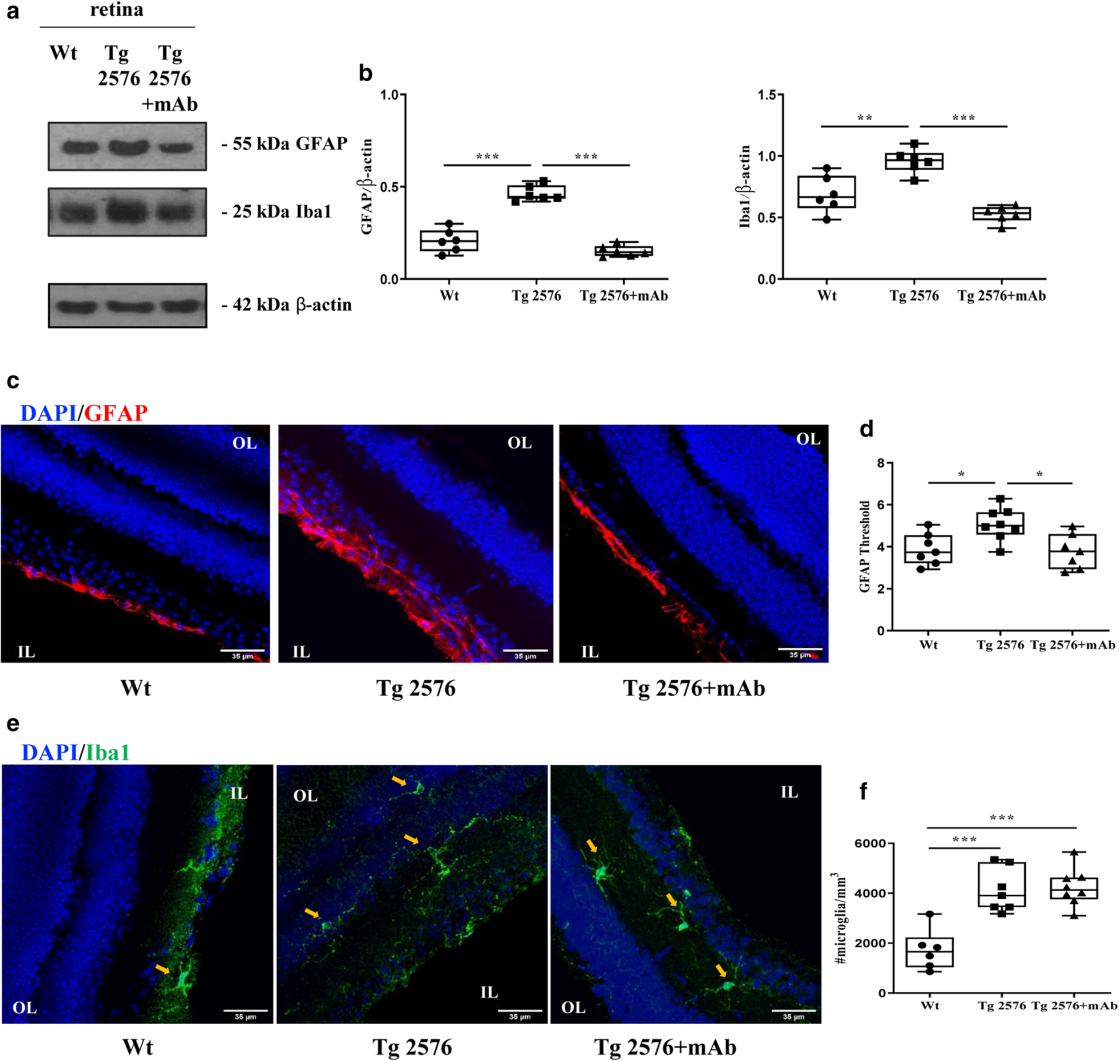
The inflammatory activation (reactive astrocytes and microglia) in retinas from AD transgenic animals is dampened by 12A12mAb treatment. Retinal homogenates extracts from animals of three experimental groups (wild-type, Tg2576 and Tg2576 + mAb) were analyzed by Western blotting for inflammatory proteins (GFAP, Iba1) (**a**). Semi-quantitative densitometric analysis (n = 6) of intensity signals (**b**) indicates lower levels of GFAP and Iba1 in Tg2576 mice + mAb compared to not-immunized counterpart. β-actin was used as loading control. Arrows on the right side indicate the molecular weight (kDa) of bands calculated from migration of standard proteins. Values are from at least three independent experiments and statistically significant differences were calculated by one-way analysis of variance (ANOVA) followed by Bonferroni’s post-hoc test for multiple comparison among more than two groups. *p* < 0.05 was accepted as statistically significant (*p < 0.05; **p < 0.01; ***p < 0.0005; ****p < 0.0001). Representative images of retinal slices from animals of three experimental groups (wild-type, Tg2576 and Tg2576 + mAb) immunolabeled with anti-GFAP antibody (red) and Hoechst for nuclei visualization (blue); Scale bar 35 μm (**c**). Quantification of GFAP area covered by fluorescent signal/field of view (*p < 0.05 Tg2576 versus wild-type; *p < 0.05 Tg2576 versus Tg2576 + mAb; n = 8 slices/3 mice for each group; One-way ANOVA, post-hoc Bonferroni test) (**d**). **e**, **f** Representative images of retinal slices from animals of three experimental groups (wild-type, Tg2576 and Tg2576 + mAb) immunolabeled with anti-Iba1 antibody (green) and Hoechst for nuclei visualization (blue); Scale bar 35 μm (**e**). Quantification of number of microglia cells in the volume of each field of view (***p < 0.0005 wild-type versus Tg2576;***p < 0.0005 wild-type versus Tg2576 + mAb; n = 8 slices/3 mice for each group; One-way ANOVA post-hoc Bonferroni test). IL: inner layer; OL: outer layer

The proper interaction between glia and neurons is known to contribute to retinal homeostasis [93]. Glial cell activation and related inflammatory responses have been previously described in retinas from human cases [17, 94, 95] and from different transgenic mouse AD models, including Tg2576, together with neurodegeneration [5, 6, 78, 96–99]. Therefore, we further examined the expression of Glial Fibrillary Acidic Protein (GFAP) and Iba1—two cell-specific markers of astrocytes and microglia, respectively—to evaluate the degree of astrogliosis and microglia infiltration under our experimental conditions. Semi-quantitative densitometry of Western blotting analyses carried out on soluble retinal extracts (Fig. 3a, b) displayed a significant upregulation of both GFAP and Iba1 immunoreactivity intensity signals in naive 6-month-old Tg2576 mice when compared with wild-type controls (***p < 0.0005; **p < 0.01, respectively). More importantly, in relation with the antibody-mediated reduction of the NH_2_htau amount in ocular samples (Fig. 1a, b) and in a similar way we previously detected in hippocampi [57], the high expression levels of both GFAP and Iba1 markers—which are known to be linked with destruction of retinal functionality [6, 10]—were strongly reduced in Tg2576 retinas following 12A12mAb immunization (***p < 0.0005 versus sham-immunized counterpart).

Confocal analysis of GFAP staining in the Tg2576 mice retina showed marked astrogliosis (measured as fluorescence intensity) localized at the level of the ganglion cell layer. Even though astrogliosis may arise also as a consequence of aging, the amount of astrocyte activation was more pronounced in the AD retina compared to controls (Fig. 3c middle and left; *p < 0.05), as quantified by fluorescence intensity in each field of view. The level of astrogliosis was significantly reduced by 12A12mAb immunization (Fig. 3c, right; Fig. 3d; *p < 0.05 vs Tg2576).

Increased microglia reactivity in the retina, visualized as positive staining for Iba1 microglial marker, revealed that this cell type was mainly present in two layers: the inner plexiform and the outer plexiform layers (Fig. 3e). Microglia cell density was increased in Tg2576 retinas compared to age matched controls (***p < 0.0005, Fig. 3e left, middle). However, while 12A12mAb immunization was able to reduce total Iba1 protein levels in retinal extracts, we did not find statistically-significant rescue of microglia cell density following treatment (Fig. 3f).

Collectively, these findings indicate that the toxic NH_2_htau peptide can participate in vivo to the pathological glial activation occurring in eyes of symptomatic Tg2576 animals and that its antibody-mediated neutralization is beneficial to the AD phenotype by exerting an overall anti-inflammatory effect, as we previously reported in their brains [57].

### Synaptic and microtubule retinal changes are mitigated and apoptosis is inhibited by i.v. 12A12mAb delivery in Tg2576 AD mice

**Fig. 4:**
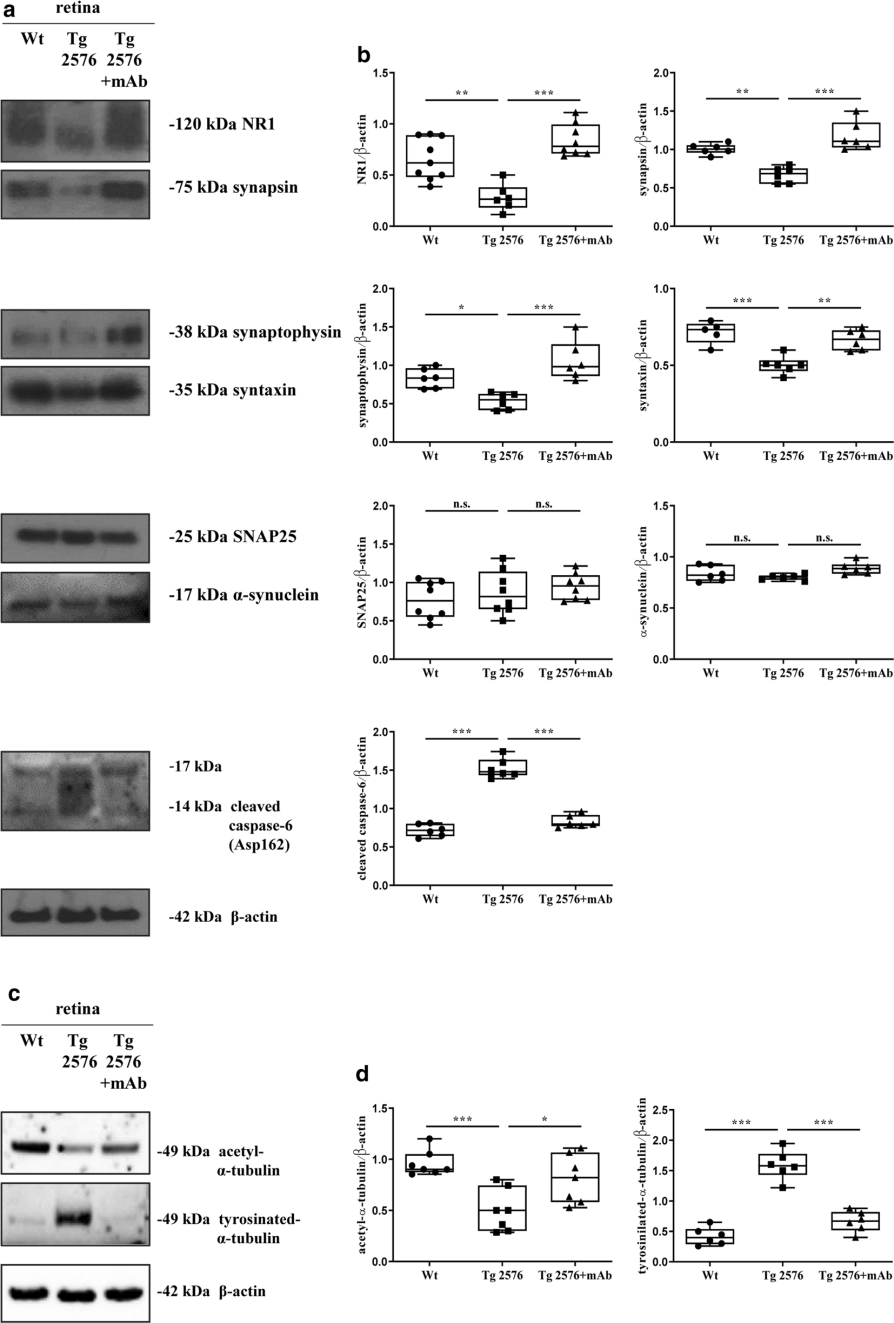
Immunotherapy with 12A12mAb mitigates the AD-associated synaptic and apoptotic changes and prevents the microtubule destabilization in retinas from diseased animals. Western blotting analyses (**a**) were carried out on equal amounts of total protein extract (50 µg) from retinas of animals of three experimental groups (wild-type, Tg2576 and Tg2576 + mAb). Immunoblots were probed with antibodies against several pre- and postsynaptic proteins—including the N-Methyl-D-aspartate (NMDA) receptor subunits 1 (NR1), synapsin I, synaptophysin, syntaxin 1, SNAP25, α-synuclein and the active (cleaved) form of caspase-6 (Asp162). Data were quantified for molecular weight size ranges for each antibody and normalized to β-actin which was used as loading control. Relative intensity of each protein was calculated and semi-quantitative densitometric analysis (n = 7) is shown (**b**). Arrows on the right side indicate the molecular weight (kDa) of bands calculated from migration of standard proteins. Statistically significant differences (see details in the main text) were calculated by one-way analysis of variance (ANOVA) followed by Bonferroni’s post-hoc test for multiple comparison among more than two groups. *p* < 0.05 was accepted as statistically significant (*p < 0.05; **p < 0.01; ***p < 0.0005; ****p < 0.0001). The functional integrity of axonal track was evaluated by probing the immunoblots with antibodies against the acetyl- and tyrosinylated-α-tubulin, as markers for stable and unstable/dynamic microtubule respectively (**c**). Data were quantified for molecular weight size ranges for each antibody and normalized to β-actin which was used as loading control. Relative intensity of each protein was calculated and semi-quantitative densitometric analysis (n = 6) is shown (**d**). Arrows on the right side indicate the molecular weight (kDa) of bands calculated from migration of standard proteins. Statistically significant differences (see details in the main text) were calculated by one-way analysis of variance (ANOVA) followed by Bonferroni’s post-hoc test for multiple comparison among more than two groups. *p* < 0.05 was accepted as statistically significant (*p < 0.05; **p < 0.01; ***p < 0.0005; ****p < 0.0001)

Reduction of synaptic connectivity is considered the earliest pathological change preceding the neuronal loss in AD subjects [100, 101] and early activation of apoptotic markers is causally associated with pathological tau truncation in AD brains [102–105]. Therefore, we evaluated the effect of NH_2_-truncation of tau on the retinal nerve terminals and the degree of cell death in 6-month-old Tg2576 mice before and after the 12A12mAb immunization. SDS-PAGE resolution of soluble extracts from retinas of the three experimental groups was analyzed with antibodies against known pre- and post-synaptic proteins, including the N-Methyl-D-aspartate (NMDA) receptor subunits 1 (NR1), synapsin I, syntaxin 1, synaptophysin, SNAP25, α-synuclein and the cleaved (Asp162) caspase-6 active form (Fig. 4a, b). Semi-quantitative densitometric analysis of immunoblots from synaptic proteins showed that, unlike SNAP25 and α-synuclein, the intensity of signals of the 120 kDa NR1, 75 kDa synapsin I, 38KDa synaptophysin and 35 kDa syntaxin 1 bands were significantly lower in transgenic mice than in littermate controls (*p < 0.05; **p < 0.01; ***p < 0.0005). Under these experimental conditions, the normal retinal expression of synaptic markers [106, 107] was strongly affected by tau truncation, in line with previous in vivo studies referring a major role of protein hyperphosphorylation in promoting the reduction of synaptophysin protein abundance during eye injury [108]. Furthermore, the retinal neurodegeneration measured as immunoreactivity of cleaved caspase-6—which is known to be activated in injured adult retinal ganglion cells [109, 110]—was higher in naive Tg2576 mice (***p < 0.0005) in comparison with wild-type controls. This finding is in line with previous investigations reporting that apoptotic signs are detected early in the eyes of 3xTg mice, another AD-relevant animal model with retinal tau accumulation and degeneration [99]. More importantly, loss in retinal synapses was largely sensitive to 12A12mAb immunization because the steady state level of synaptic markers appeared to be significantly upregulated in the Tg2576 group following antibody delivery in comparison with the not-injected counterpart (**p < 0.01; ***p < 0.0005). Likewise, the activation of caspase-6 effector found in transgenic retinas was significantly decreased following 12A12mAb injection (***p < 0.0005).

Cytoskeleton destabilization followed by impairment in axonal transport is a crucial factor contributing to synaptic deterioration in AD. Tau, which belongs to the family of microtubule-associated proteins (MAP), is crucially involved in the maintenance of microtubule assembly and integrity [53]. Therefore, we investigated the dynamic state of microtubule network in retinal homogenates from the three experimental groups by Western blotting (Fig. 4c, d) with antibodies against acetyl (stable)—and tyrosinylated (unstable)-α-tubulin, which are considered two reliable markers for stable and unstable/dynamic microtubules respectively. Interestingly, in Tg2576 AD mice, the immunoreactivity level of stable acetylTub was strongly decreased when compared with their littermate controls (***p < 0.0005) but significantly restored after injection with 12A12mAb (*p < 0.05). Consistently, an inverse trend was detected for the tyrTub-signal whose increment in naive transgenic animals (***p < 0.0005) was significantly downregulated up to physiological baseline after administration of 12A12mAb (***p < 0.0005). These results are in good agreement with the 12A12mAb-dependent reciprocal changes in AT8/Tau-1 intensity pattern (Fig. 2a, b) that we detected under the same experimental conditions. These findings suggest that the treatment with antibody is more likely to normalize the cytoskeleton dynamics via site-specific phosphorylation of endogenous murine tau, which is critically involved in modulating the assembly/polymerization of the microtubule network.

Taken together, these results demonstrate that, in addition to stimulating the inflammatory response, the toxic NH_2_htau peptide can also impinge on retinal synaptic proteins—likely as a consequence of the microtubule breakdown—and on the extent of local caspase-dependent cell death in Tg2576 AD model.

### Intravenous delivery of 12A12mAb partially normalizes the neurochemical alterations in retinas of Tg2576 AD mice

Aberrant excitatory activity and compensatory remodeling of inhibitory hippocampal circuits, which lead to neural network dysfunction, play a crucial role in cognitive deficits in hAPP-expressing mice including Tg2576 [111, 112] and, possibly, also in humans suffering from AD. In the inner retina, the functional circuitry is mainly controlled by cooperative glutamatergic, cholinergic and GABAergic mechanisms involving the amacrine cells which establish glutamatergic synapses with bipolar cells in Outer Plexiform Layer (OPL) and Inner Plexiform Layer (IPL) and receive GABAergic and cholinergic inputs from other amacrine cells [113]. Amacrine cells, together with horizontal cells, modulate neurotransmission along the synaptic axis, including photoreceptors (cones and rods), bipolar and ganglion cells whose axons (optic nerve) convey the signal to the visual cortex.

**Fig. 5:**
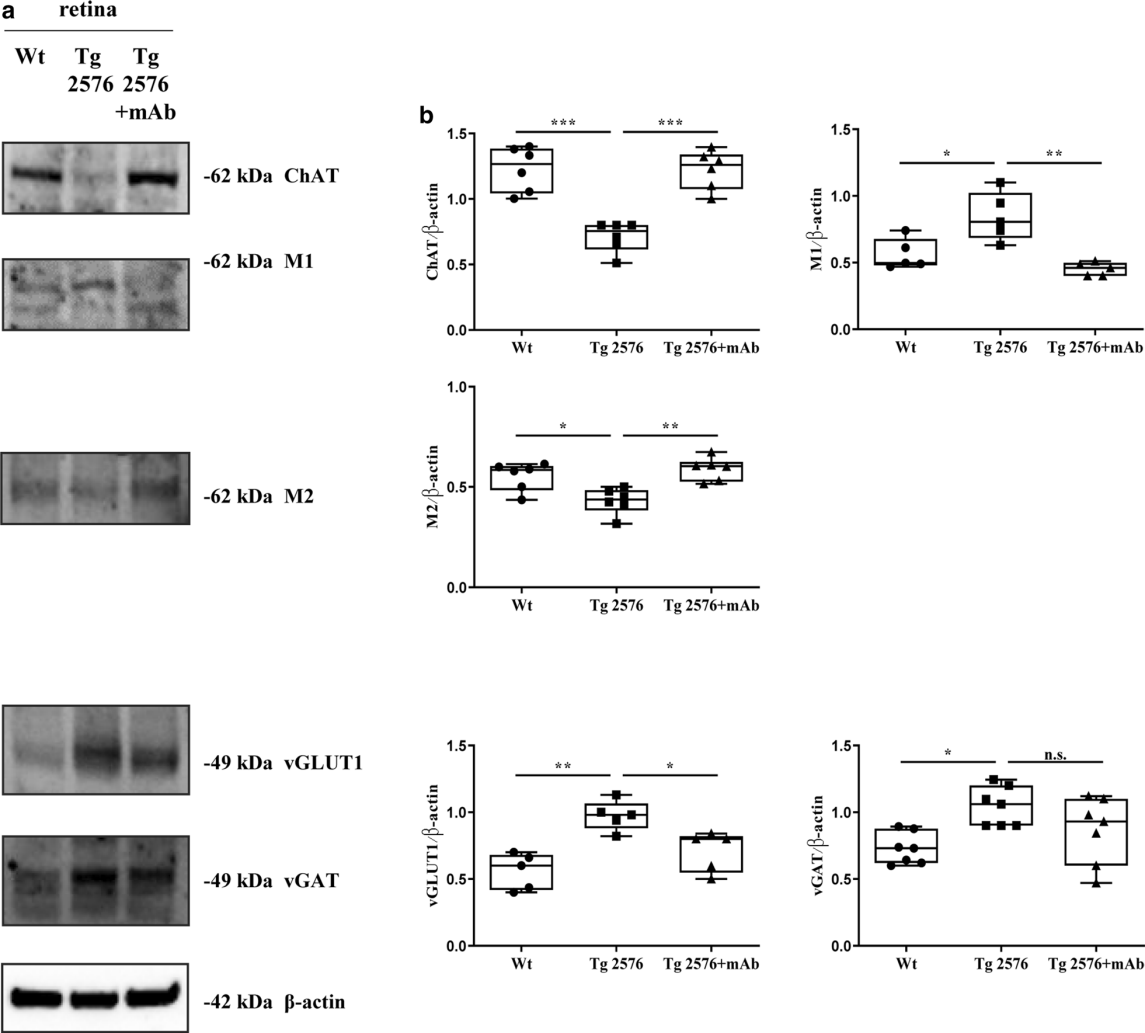
Neurochemical abnormalities occurring in the retinas from Tg2576 AD mice are responsive to treatment with 12A12mAb. Representative images of Western blotting analyses (**a**) carried out on equal amounts of total protein extract (50 µg) from retinas of animals of three experimental groups (wild-type, Tg2576 and Tg2576 + mAb). Filters were probed with antibodies against the Choline acetyltransferase (ChAT), the muscarinic acetylcholine receptor (M1), the vesicular GLUtamate transporter1 (vGLUT1), the vesicular GABA transporter (vGAT). Data were quantified for molecular weight size ranges for each antibody and normalized to β-actin which was used as loading control. Relative intensity of each protein was calculated and semi-quantitative densitometric analysis (n = 6) is shown (**b**). Arrows on the right side indicate the molecular weight (kDa) of bands calculated from migration of standard proteins. Statistically significant differences were calculated by one-way analysis of variance (ANOVA) followed by Bonferroni’s post-hoc test for multiple comparison among more than two groups. *p* < 0.05 was accepted as statistically significant (*p < 0.05; **p < 0.01; ***p < 0.0005; ****p < 0.0001)

Thus, we investigated whether the ocular changes in synaptic protein expression were also associated with concomitant neurochemical alterations in 6-month-old Tg2576 mice and in a 12A12mAb-dependent manner. To these aims, Western blotting analysis was carried out on retinal homogenates from the three experimental groups with antibodies against Choline AcetylTransferase (ChAT), Muscarinic acetylcholine receptor (M1), vesicular GLUtamate Transporter1 (vGLUT1) and vesicular GABA Transporter (vGAT) (Fig. 5a). Interestingly, a statistically significant (***p < 0.0005) reduction in 68 kDa ChAT signal was detected in naive Tg2576 when compared to their littermate wild-type controls accompanied by an inverse, likely compensative, increase in 52 kDa M1 immunoreactivity (*p < 0.05). Strikingly, 12A12mAb immunization restored the protein expression levels of these two cholinergic markers from transgenic AD mice up to physiological baseline (***p < 0.0005; **p < 0.01). A similar trend was detected for the glutamatergic vGLUT1 signal, whose upregulation was found to be pronounced (**p < 0.01) in the untreated transgenic group but significantly diminished after i.v. injection with 12A12mAb (*p < 0.05). On the contrary, 12A12mAb delivery appeared to be ineffective in balancing the intensity of vGAT, a GABAergic marker found to be slightly increased in Tg2576 AD animals (*p < 0.05), because no significant difference was found when the untreated transgenic group was compared to its antibody-treated counterpart (n.s. = not significant).

Collectively, these results show that the pathological accumulation of the toxic NH_2_htau peptide in eyes of Tg2576 mice is associated with changes in cholinergic, glutamatergic and GABAergic neurotransmission in a way resembling the disruption in the excitatory-inhibitory balance occurring in the vulnerable circuitries of their AD-affected brains.

### Mitochondrial metabolism and ATP production in the retinas of Tg2576 AD mice are restored by treatment with 12A12mAb

Mitochondrial perturbations and axonopathy are prominent features of human tauopathies, including AD [114, 115]. Similarly to the brains, the accumulation of pathological tau impairs the mitochondrial metabolism and axonal transport in 3xTg mouse retinas [63] and in a model of diabetic retinopathy [108].

In view of these considerations, by Western blotting analysis on retinal protein extracts from three experimental groups, we investigated the mitochondrial status with antibodies against several key structural and functional proteins, including the Optic Atrophy Type 1 (OPA1), a dynamin-related GTPase controlling the organelle dynamics (mitophagy), the mitochondrial outer membrane translocase 20 (TOMM 20) and the Voltage-Dependent Anion-selective Channel 1/porin (VDAC 1), which allow for the conductance of molecules into and out of the organelle, the Manganese SuperOxide Dismutase (MnSOD), which is an antioxidant enzyme with reactive oxygen species (ROS) scavenging activity and the Cytochrome c (Cyt c), which catalyzes the last steps in the ETC for ATP synthesis.

**Fig. 6:**
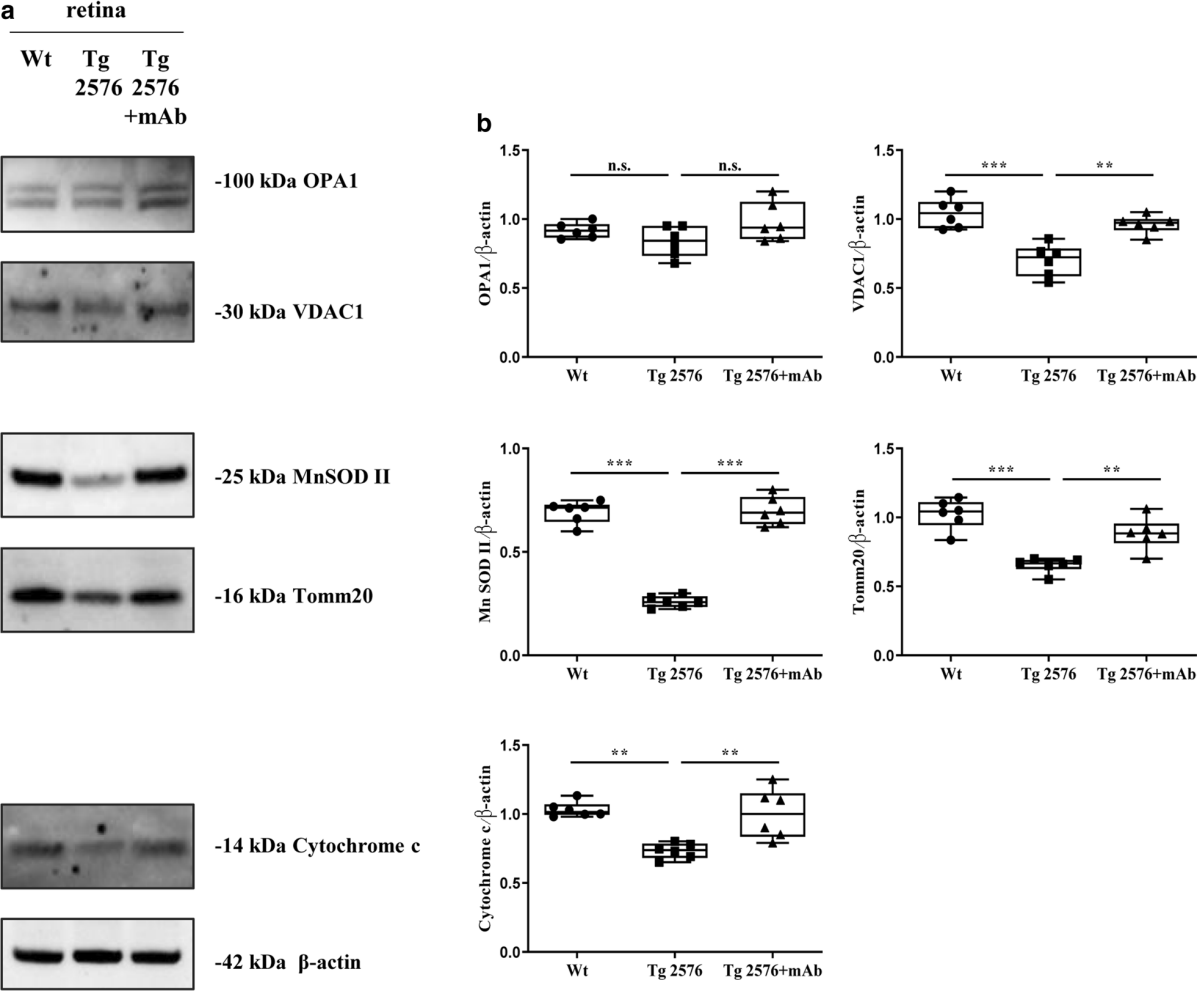
Targeting of tau truncation by i.v. 12A12mAb injection protects against the alterations in expression level of mitochondrial proteins occurring in the retinas from Tg2576 AD mice. **a**, **b** Equal amounts of total protein extract (50 µg) from retinas of animals of three experimental groups (wild-type, Tg2576 and Tg2576 + mAb) were analyzed by SDS-PAGE with specific antibodies against several mitochondrial markers, including the Optic Atrophy Type 1 (OPA1), the outer membrane translocase 20 (TOMM 20), the Voltage-Dependent Anion-selective Channel 1/porin (VDAC 1), the Manganese SuperOxide Dismutase (MnSOD) and the Cytochrome c (Cyt c). Data were quantified for molecular weight size ranges for each antibody and normalized to β-actin which was used as loading control. Relative intensity of each protein was calculated and semi-quantitative densitometric analysis (n = 6) is shown (**b**). Arrows on the right side indicate the molecular weight (kDa) of bands calculated from migration of standard proteins. Statistically significant differences were calculated by one-way analysis of variance (ANOVA) followed by Bonferroni’s post-hoc test for multiple comparison among more than two groups. *p* < 0.05 was accepted as statistically significant (*p < 0.05; **p < 0.01; ***p < 0.0005; ****p < 0.0001)

Strikingly, semi-quantitative densitometry of signal intensities from immunoblots (Fig. 6a, b) showed that the steady-state expression levels of VDAC 1, MnSOD, TOMM 20, and Cyt c were downregulated in Tg2576 AD mice in comparison with their littermate wild-type controls (***p < 0.0005; **p < 0.01) but significantly rescued after i.v. administration of 12A12mAb (***p < 0.0005; **p < 0.01). Conversely, no significant change was detected among the three experimental groups in the immunoreactivity of OPA 1 whose genetic knockdown is linked to Autosomal Dominant Optic Atrophy (ADOA), a hereditary disorder characterized by progressive loss of vision following alteration in mitochondrial network [116].

**Fig. 7:**
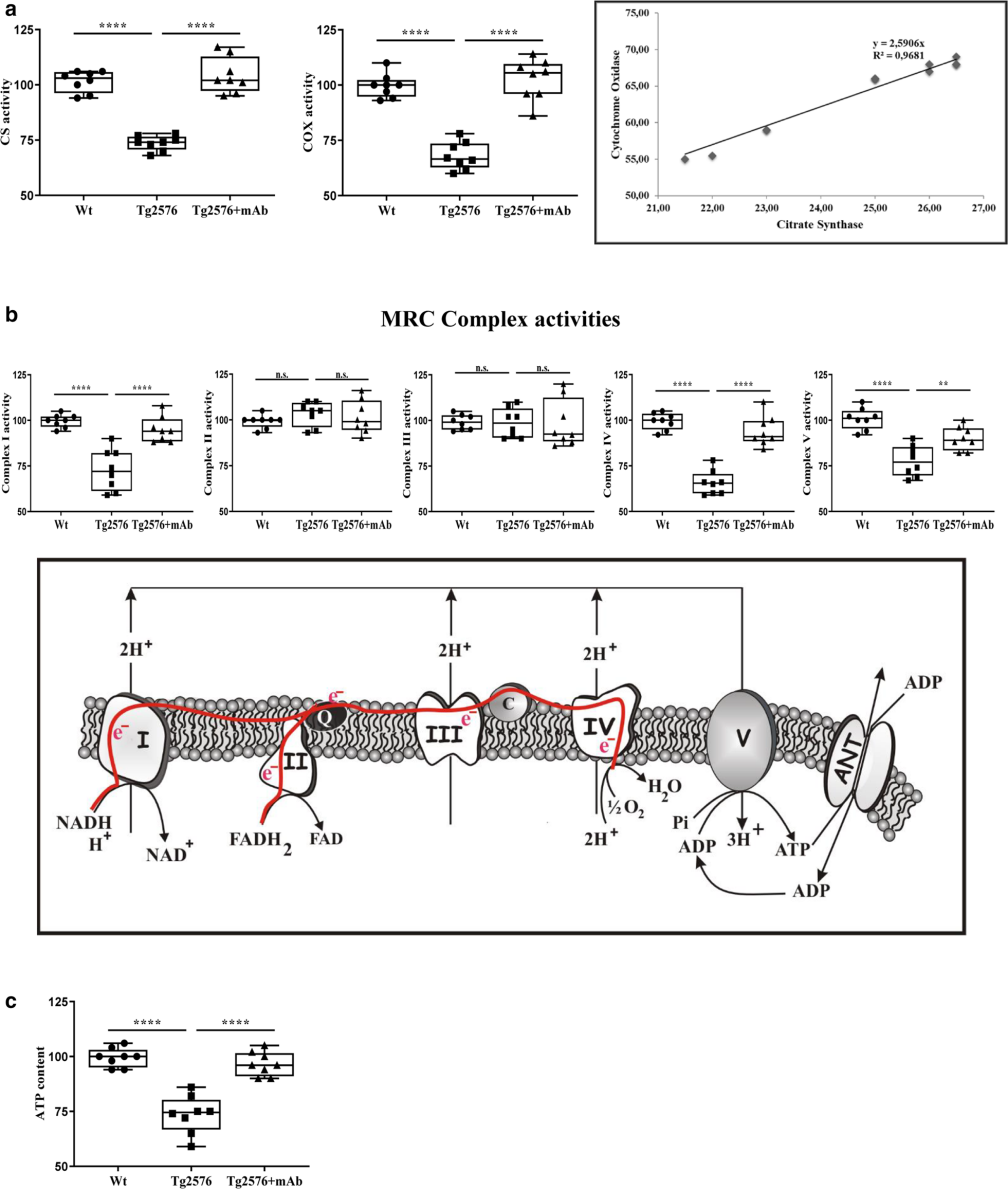
Mitochondrial ATP-production is significantly recovered in retinas from Tg2576 AD mice following passive immunization with 12A12mAb. Analyses of mitochondrial functionality (n = 8) carried out on homogenates from retina specimens are shown. **a** Citrate synthase (CS) and cytochrome oxidase (COX) specific activities measurements. **b** MRC complex activities: the activities of complex I (NADH:ubiquinone oxidoreductase), complex II (succinate:ubiquinone oxidoreductase), complex III (cytochrome c reductase), complex IV (cytochrome c oxidase) and complex V (ATP synthase). A schematic representation of OXPHOS system is also reported in the inset. **c** The cellular ATP content. Statistically significant differences were calculated by one-way analysis of variance (ANOVA) followed by Bonferroni’s post-hoc test for multiple comparison among more than two groups. *p* < 0.05 was accepted as statistically significant (*p < 0.05; **p < 0.01; ***p < 0.0005; ****p < 0.0001)

To further deepen these findings, we performed analyses of the mitochondrial content, the repiratory chain activity and the ATP content (Fig. 7a–c). In detail, the mitochondria content as well as mitochondrial respiratory capacity were estimated in total homogenates from animals’ retina specimens by measuring spectrophotometrically the activities of citrate synthase (CS) and citochrome oxidase (COX), respectively. Of note, CS is an enzyme of the Krebs cycle, encoded in cell nucleus, synthesized on cytoplasmic ribosomes and transported into the mitochondrial matrix. It is one of the best indicators for mitochondrial content in tissues [117], since it is considered as a stably-expressed enzyme in a specific tissue [118–120]. COX activity, an enzyme controlled by both nuclear and mitochondrial genomes, catalyzes a step in the mitochondrial electron transfer chain (ETC) and was chosen as the reference for the oxidative phosphorylation (OXPHOS) system activity because this enzyme constitutes the last step in the mitochondrial respiratory chain (MRC) and, likely, it is limiting its electron flux [65]. Results displayed large differences in the specific activities (normalized by the total tissue homogenate protein content) of CS and COX occurring among ocular specimens. Consistent with the notion that cells losing mitochondrial mass are unable to efficiently meet bioenergetic needs [121], a positive and highly significant correlation (R^2^ = 0.968) was found between CS and COX activities per cell indicating that the samples having the lowest/highest COX activity also had the lowest/highest CS activity.

As shown in Fig. 7a, both CS and COX activities were found to decrease in Tg2576 AD mice when compared with littermate wild-type controls (****p < 0.0001) and significantly rescued after i.v. 12A12mAb delivery (****p < 0.0001). We then investigated bioenergetic features of the OXPHOS system (Fig. 7b), by determining the individual activities of mitochondrial complexes I–V composing the mETC and the cellular ATP levels. When compared with wild-type controls, the analysis of the five MRC complexes in retinas from Tg2576 mice revealed a significant reduction in the activities of complexes I and IV (****p < 0.0001) which were recovered by 12A12mAb injection nearly up to the physiological wild-type control baselines (****p < 0.0001). On the contrary, no significant difference in the activity of complex II and complex III was detected among the three experimental groups (*p* > 0.05). Besides, the ATP synthase (complex V) activity was also markedly downregulated in Tg2576 samples (****p < 0.0001) and restored, althought to a lesser degree, following 12A12mAb immunization in statistically-significant manner (****p < 0.0001). Consistent with a strong impairment of OXPHOS in Tg2576 AD mice, the overall content of ATP—which is generally considered a good indicator of the cellular healthy conditions [122]—was drastically lower in AD transgenic mice than in their littermate wild-type controls (****p < 0.0001) with a nearly complete rescue to control values following 12A12mAb immunization (****p < 0.0001) (Fig. 7c). In combination with biochemical data on synaptic expression and cytoskeleton integrity (Fig. 4a–d), these results strongly support the hypothesis that in Tg2576 AD mice the endogenously-generated NH_2_htau fragment can impinge on retinal degeneration, and possibly on animals’ visual disability, via direct and/or indirect changes of mitochondrial metabolism: (1) by promoting the microtubule breakdown which causes impairment of axonal trafficking, including mitochondria and synaptic vesicles, towards the terminal ends; (2) through inhibition of OXPHOS energy production which further exacerbates the synaptic starvation and derangement.

These findings confirm and further extend our previous studies reporting a noxious effect exerted by NH_2_htau fragment on the normal physiology of neuronal mitochondria in AD [52, 53], demonstrating that its 12A12mAb-mediated in vivo clearance could be also exploited to mitigate the visual deficits associated with mitochondrial dysfunction due to the retinal accumulation of pathogenetic tau species [63, 108].

## Discussion

It is now largely recognized that the retina faithfully mirrors some pathological events occurring in the brain [14]. The importance of studying the ocular manifestations in AD pathology stems from the evidence that the retina, being a simple and accessible experimental system, can be used as a molecular proxy to diagnose early degenerative alterations in the brain before the neuronal loss is irreversible and/or to refine therapeutic strategies [1, 123–125]. Alterations in APP/Aβ and tau metabolism, mitochondrial dysfunctions, defects in axonal transport, synaptic remodeling, neuroinflammation are all pathophysiological changes detected in both AD and retinal decay [14, 126], enabling thus the interchange of knowledge in terms of underlying pathogenetic mechanisms and therapeutic intervention [16].

The current study unveils for the first time that, in addition to Aβ deposits and tau-positive aggregates [16, 18, 127], the aberrant tau cleavage is another common pathological feature shared by AD-affected neuroretina and brain. Consistent with this finding, we discover that truncation at the N-terminal domain of tau is closely linked to degeneration both of the retinas and vitreous bodies from 6-month-old Tg2576 transgenic mice which overexpress a mutant form of amyloid precursor protein (APP), APPK670/671L, linked to early-onset familial AD. This well-established animal model carrying endogenous murine not-mutated tau, represents an ideal model to study the AD-associated changes, both in the retina and in the brain. Indeed, Tg2576 mice show progressive retinal ganglion cell loss and visual disabilities, features that develop together with other known ocular pathologically-relevant changes such as APP/Aβ misprocessing [128], tau hyperphosphorylation/oligomerization [63, 78], gliosis [129, 130], loss of synaptic proteins, alteration in mitochondrial functions and neuronal death [1, 16, 108]. Alterations of tau metabolism (aggregation, hyperphosphorylation) have been previously associated with deterioration of retinal structures in AD subjects and tauopathies of animal models [7, 8, 15, 77, 78, 80]. Increased susceptibility to excitotoxic injury and changes in pathways of neurotrophic factor signal transduction are detected in the retina of the P301S mutant human tau transgenic animals [131, 132]. Consistently, genetic reduction of tau accumulation occurring in Retinal Ganglion Cells (RGC) and optical nerves of 3-month-old 3xTg-AD mice significantly improves cell density and functionality [63]. Interestingly, tau-mediated pathogenic mechanisms are also involved in other age-related oculopathies, as shown by decreased tau levels in the retina [133] and increased levels of tau in the vitreous of patients bearing glaucome and diabetic retinopathy [134]. In this context, our in vivo results linking the tau truncation to pathological ocular changes occurring in symptomatic Tg2576 mice offer new insights into the tau-dependent events characterizing the retina and vitreous humor in AD conditions. Besides, our biochemical evidence show that upregulation in the immunoreactivity of the APP695 isoform and AT8 phospho-tau can be already detected in eyes of these mice at 6 months of age, further extending previous in vivo studies on older animals (14/18-month-old) [6, 9]. With regard to Western blotting analysis on the expression levels of selective synaptic proteins, the alterations are partially in contrast with other immunohistological investigations showing that the density of PSD95 and synaptophysin—two markers of postsynaptic and presynaptic integrity—are unchanged in Tg2576 retinal sections up to 14 months of age [72]. We think that this discrepancy could be due to the use of experimental procedures with different sensitivity (biochemical versus histologic methods), the dissimilarity between the analyzed retinal regions and the potential occurrence of compensative age-related changes in dendritic complexity which can, indirectly, preserve the synaptic number and, then, the synaptic density. Of note, in this study [72], only female Tg2576 are used while, in another one [135], only male Tg2576 are employed with no apparent differences in retinal degeneration among the two sexes. Moreover, the detrimental action we found on retinal synaptic and mitochondrial functions exerted in vivo by tau truncation fits well with investigations referring a pivotal role of AD-like site-specific hyperphosphorylation at serine 396 (S396) and 404 (S404) and threonine 205 (T205) and 231 (T231) of protein, in causing the visual deficits associated with diabetic retinal neurodegeneration [108]. It’s also worth noting that, in this mutated APP-overexpressing genetic background, we are unable to discriminate between the direct and indirect effects induced by in vivo neutralization of the toxic NH_2_htau fragment following 12A12mAb immunization. Besides, a tight connection between pathological tau and APP/Aβ dysmetabolism would drive the disease pathway through truncated N-tau with further increasing APP/Aβ levels along a self-perpetuating destructive cycle [136]. Therefore, the present study strengthens and further extends our previous results [57] showing that: (i) an interplay occurring between APP/Aβ misprocessing and post-translational modifications of endogenous murine tau is more likely to drive the AD-like neurodegeneration in APP-expressing Tg2576 animal model and (ii) reducing tau pathology via the Aβ pathway can be a good therapeutic strategy, both in the retina and brain.

From a translational point of view, an interesting finding of this study is the evidence that the pathogenic N-terminal truncated 20–22 kDa tau peptide is expressed at high levels both in different ocular structures (retina and vitreous body) and in brain parenchyma of Tg2576 AD mice [57]. Considering that the eye is structurally less complex and more accessible than the brain, the current observation features the retina and vitreous humor—which can provide indirect information on retinal microenvironment [92, 126]—as reliable sources of clinically-predictive, tau-based ocular biomarkers of AD cerebral neurodegeneration. Consistently, by using a qualitative cross-sectional approach, den Haan et al. [15] have recently reported that statistically-significant differences in tau hyperphosphorylation (AT8, AT100, AT270) are distinctly visible in the retina of AD autoptic specimens in comparison with age-matched not-demented controls. In this framework, since the retinal staining by 12A12mAb is mainly confined to the Ganglion Cell Layer (GCL, the output neurons of the eye) as shown for hyperphosphorylated tau in eyes of tauopathy transgenic models [79] and AD cases [15], the pathogenic N-truncated tau could be exploited as feasible and accessible candidate for the visual exploration of AD pathology by in vivo-imaging techniques [124, 137]. Furthermore this tau peptide, as demonstrated for APP/Aβ-derivates [92], appears to follow in ocular fluids of the Peripheral Nervous System (PNS) a similar pattern to that observed in the Central Nervous System (CNS) where it is primarily generated in neurons and released into the CerebroSpinal Fluid (CSF). To this regard, we and other reasearch groups have reported that this tau-derived soluble specie(s) accumulates at human AD presynaptic terminals [53, 54, 64, 73] and is present in CSF from patients suffering from AD and other related tauopathies [52, 138]. Besides, the future employment of not-invasive retinal imaging and eye-based protein biomarkers for early diagnosis and monitoring therapeutic efficacy is widely fostered by the recent finding that the quantification of Aβ1-40/1–42 and total tau levels in the vitreous, an ocular fluid which is considered to be a direct indicator of the underneath suffering retina, has predictive clinical utility in the clinical practice of AD [76].

**Fig. 8:**
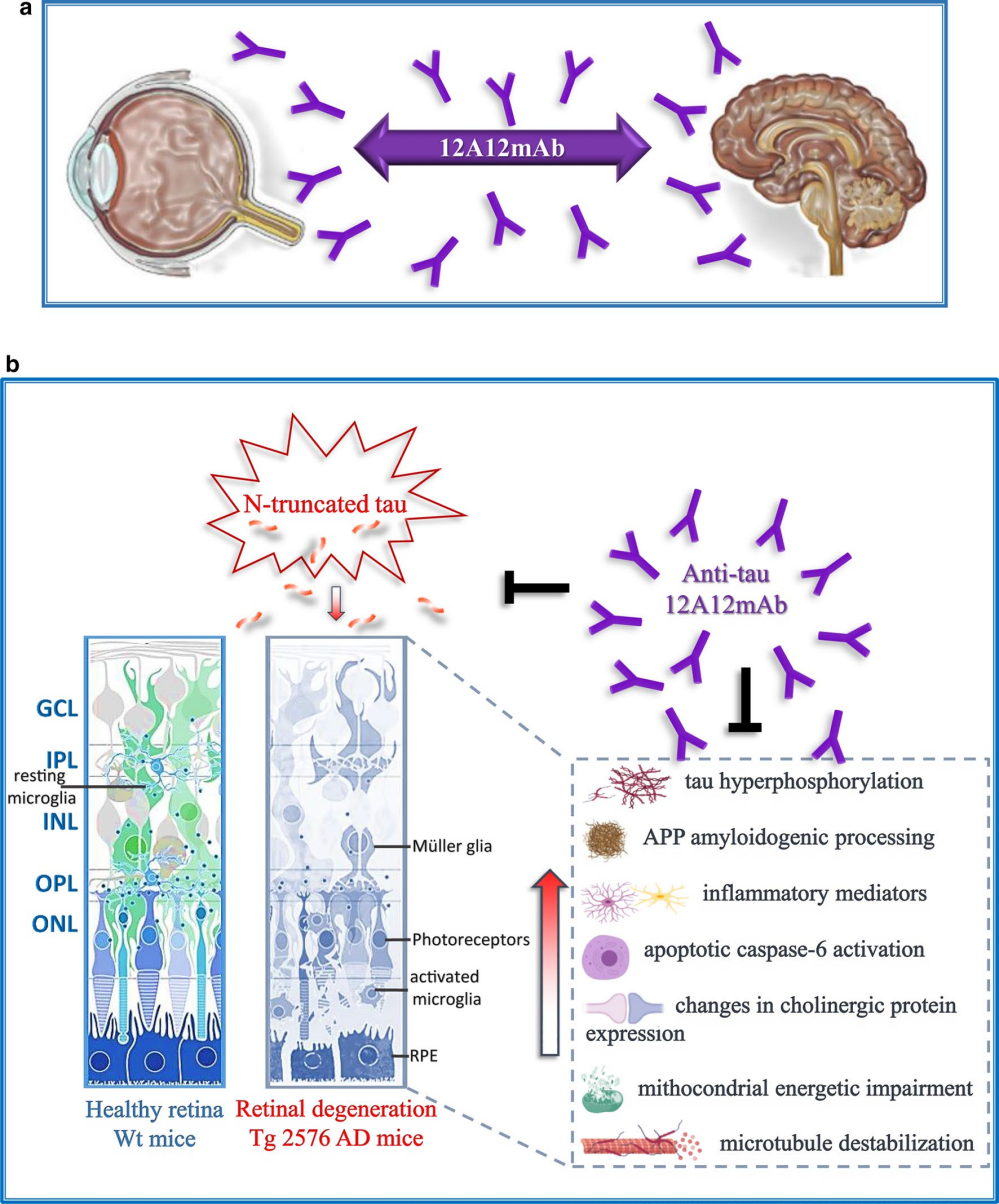
12A12mAb as novel tau-directed immunotherapeutic tool for the clinical treatment of retinal degeneration associated with AD. **a** A graphical illustration showing the parallel neuroprotective effects of 12A12mAb on both aging brain [136] and eye. **b** Schematic representation showing the 12A12mAb-mediated neutralization of the AD-relevant N-truncated tau fragment residing in ocular structures and its beneficial actions on alterations associated with visual impairment in Tg2576 animal model

The preclinical results from this study show that the neuroprotective effects offered by 12A12mAb delivery on the eyes from Tg2576 AD model (Fig. 8) are paralleled by the contextual improvement in hippocampal-dependent cognitive functions owing to antibody-mediated paired reduction of the 20–22 kDa tau fragment [57]. This observation demonstrates that the production and/or the clearance of this AD-relevant pathogenic tau specie(s) [139], either in the neuroretina and in the brain, are tightly interwined and both strongly responsive to immunotherapy. Consistent with this finding, the in vivo 12A12mAb-mediated neutralization of the 20–22 kDa tau fragment in eyes occurs, as in the brain [57], in the absence of reactive gliosis which, on the contrary, appears to be a harmful byproduct of the mechanism of action of Aβ-directed antibodies. However, the evidence that delivery of 12A12mAb dampens the local retinal inflammation (as evidenced by astroglial and microglial activation) suggests that a more complex neuron-glia interplay occurs in vivo since the reduction of Aβ retinal deposits, following vaccination with Aβ oligomer antigens, is shown to provoke in Tg2576 mice an opposite exacerbation of microglial infiltration and astrogliosis followed by disruption of retinal architecture [6]. Besides, these findings confirm previous investigations reporting that the genetic reduction of tau expression by intravitreal injection of targeted siRNA, ameliorates the axonal transport [63], the synaptic and mitochondrial defects [108] in 3xTg AD mice and in a High-Fat Diet (HFD)-induced animal model of diabetic retinopathy, leading to improvement of their visual abnormalities [108]. It is also worth noting that 12A12mAb is a cleavage-specific neoepitope antibody which selectively engages the 20–22 kDa neurotoxic form of tau [57], prospecting thus its safe administration in human beings in the absence of deleterious “loss of function”of the physiological full-length protein [140–143]. Our in vivo study is also consistent with the significant protection afforded by systemic administration of an Aβ-targeting specific antibody in a model of age-related macular degeneration (AMD) [81, 144]. APOE4-targeted replacement mice fed with a High Fat Cholesterol (HFC)-enriched diet present Aβ-containing deposits in the retinal pigmented epithelium (RPE) and deficits in the electroretinographic response, which are indicative of an impaired visual function. Ding et al. [81, 144] report that the passive immunotherapy by means of i.v. delivery of an antibody targeting the C-termini of Aβ40 and Aβ42 is able to significantly reduce ocular Aβ deposits in APOE4-HFC mice and, then, to preserve retinal function through a mechanism which is consistent to the peripheral sink hypothesis [145]. Alternatively, the glymphatic pathway of CSF—which enters the optic nerve via spaces surrounding blood vessels bordered by AQuaPorin (AQP)4-positive astrocytic end-feed [146–148]—could be also taking part in vivo in the antibody-mediated draining of pathological tau. The effectiveness of 12A12mAb on retinal decay of Tg2576 mice is also confirmed by the experimental evidence that the microtubule stability, the amount and metabolic state (evaluated on both the activities of respiratory chain and energy production) of mitochondria are markedly recovered after its in vivo systemic administration, nearly up to their physiological baselines. Relevantly, post-mitotic neurons have reduced glycolytic capacity and, then, strongly rely for energy production on mitochondria which are largely abundant and crucial for the survival of in RGCs endowed with great metabolic demand [149]. An interesting study [150] has recently reported parallels between retinal and brain pathology and in response to immunotherapy with Glatiramer acetate, an FDA‐approved drug which promotes microglial-mediated Aβ clearance in old APPSWE/PS1∆E9 ADtg mice. In this study, paired brains and eyeballs were collected at the end of the last injection and processed for biochemical and morphological analyses. We have immunized animals under the identical/overimposable experimental conditions (age, sex, antibody dosage, administration route, time of treatment and so on) that we previously carried out in analyzing their brain tissues [57]. As a matter of fact, although brain and eye are not part of the same animal, we have detected a frank correlation between retinal and hippocampal pathology and in response to immunotherapy. Finally, here we provide biochemical and morfological assessments and evaluation of mitochondrial metabolic activity and ATP production. We neither monitored the retinal activity by analysis of b-wave ElectroretinoGrams (ERGs) and nor performed the visual acuity performance test on the three experimental groups (wild-type, Tg2576, Tg2576 + mAb), but these functional parameters are under current investigation.

## Conclusions

This preclinical study, carried out on the well-established AD-like Tg2576 animal model indicates that the assessment of retinal tau truncation can be reliably used to diagnose and monitor brain pathology and cognitive status before neuronal loss becomes irreversible. In addition, these data provide for the first time the feasibility of tau-directed immunotherapy in ameliorating both cerebral and extracerebral manifestations associated in vivo with AD pathology.

## Data Availability

All the data used and/or analyzed for the current study is contained in the article. All other datasets are available from the corresponding author upon reasonable request.

## Abbreviations

AD: Alzheimer’s Disease
Aβ: Amyloid β-protein
NFT: NeuroFibrillary tangles
MCI: Mild cognitive impairment
mAb: monoclonal Antibody
LTP: Long term potentiation
APP: Amyoid precursor protein
CSF: CerebroSpinalFluids
GCL: Ganglion cell layer
RGC: Retinal ganglion cells
AMD: Age-related macular degeneration
MMSE: Mini-mental state exam
GFAP: Glial fibrillary acidic protein
NMDA: N-Methyl-D-aspartate
NR1: Receptor subunits 1
ChAT: Choline acetyltransferase
M1: Muscarinic acetylcholine receptor
vGLUT1: Vesicular GLUtamate transporter1
vGAT: Vesicular GABA transporter
TOMM20: Outer membrane translocase 20
VDAC 1: Voltage-dependent anion-selective channel 1/porin
Cyt c: Cytochrome c
COX I: Cytochrome c OXidase subunit 1
acetylTub: Acetyl-α-tubulin
tyrTub: Tyrosinylated-α-tubulin
ERGs: Electroretinograms
PNS: Peripheral nervous system
CNS: Central nervous system
HFD: High-fat diet
HFC: High fat cholesterol
